# Spatial Estimation of PM_2.5_ Exposure and its Association with Asthma Exacerbation: A Prospective Study in Thai Children

**DOI:** 10.5334/aogh.3513

**Published:** 2022-03-08

**Authors:** Kornnasa Chankaew, Ratchaneewan Sinitkul, Wiparat Manuyakorn, Koonkoaw Roekworachai, Harutai Kamalaporn

**Affiliations:** 1 Faculty of Medicine, Ramathibodi Hospital, Mahidol University, TH; 2 Nuffield Department of Medicine, University of Oxford, UK; 3 Mahidol-Oxford Tropical Medicine Research Unit, Faculty of Tropical Medicine Mahidol University, TH; 4 Toxicology Department, UK Health Security Agency, Harwell Campus, Chilton, Oxfordshire, UK; 5 Nakornping Hospital, Chiang Mai, TH

## Abstract

**Background:**

The acceptable fine particulate matter (PM_2.5_) level in Thailand is double the recommendation of the World Health Organization. It is necessary to have an accurate measure of PM_2.5_ exposure and its association with health problems in vulnerable groups such as asthma exacerbation in Thai children to urge the Clean Air Act in Thailand, which is currently in the process of revision.

**Objective:**

To study the association between PM_2.5_ exposure and asthma exacerbation in children living in Bangkok Metropolitan Region and Chiang Mai Province.

**Methods:**

A pilot prospective observational study was conducted at the Chest and Allergy clinic at Ramathibodi Hospital, Mahidol University, Bangkok and at the Chest Clinic at Nakornping Hospital, Chiang Mai, Thailand, from June 2020 to February 2021. Children with asthma, aged 5–18 years old, were recruited. Respiratory symptoms, including cough, chest tightness, dyspnea or wheezing, peak expiratory flow rate, and asthma exacerbation, were recorded twice daily by caregivers. Estimated average daily PM_2.5_ exposure levels were calculated using ArcGIS® at exacerbation day, three days before exacerbation (lag day 3), and 7 days before exacerbation (lag day 7). Regression analysis was applied to examine the association between PM_2.5_ exposure and asthma exacerbation.

**Findings:**

Seventy asthmatic patients were enrolled. The median age was 9.7 (IQR 5–18) years old. There were 53 respiratory symptoms, 5 admissions, and 1 intensive care unit admission. Daily PM_2.5_ levels above 12 mcg/m^3^ (the US cut-off level for the sensitive group) has higher sensitivity to detect asthma exacerbation compared to Thai cut-off level for the sensitive group (37 mcg/m^3^) (sensitivity 98.2% vs 32.1%). The average daily PM_2.5_ level exposure at lag day 3 in the exacerbation vs the non-exacerbation group was 27.5 and 13.6 mcg/m^3^ (p < 0.01), respectively. The daily PM_2.5_ level at lag day 3 was also correlated with an acute asthmatic attack (r = 0.62, p < 0.01) with the 0.2 events increasing of asthmatic exacerbation every 10 mcg/m^3^ of increment of daily PM_2.5_ level.

**Conclusions:**

Our findings suggest that asthmatic children are sensitive to daily PM_2.5_ levels above 12 mcg/m^3^. Exposure to high daily PM_2.5_ levels can lead to asthma exacerbation within three days. Further participant recruitment is needed to emphasize this association and establish the national data.

## Background

Air pollution is an important problem around the world. Over 90% of the world’s population live in places where air pollution levels exceed the limits of the World Health Organization (WHO) guidelines. Fine particulate matter (PM_2.5_) exposure was estimated to be associated with 4.2 million premature deaths and 103 million disability-adjusted life-years in 2015 [1]. It leads to devastating morbidities and mortality such as cardiovascular diseases, immune dysfunction, obesity, gene mutations, premature deaths, and especially respiratory diseases [2,3,4,5,6,7,8,9,10]. These health problems have grown rapidly over the past 3 decades. Without any effective policies, the number of deaths due to ambient air pollution will double by 2050 [10]. Epidemiological and clinical studies have shown that exposure to air pollution such as particulate matter, ozone, nitrogen dioxide, and sulfur dioxide is a crucial risk factor for poor asthma outcomes [11,12]. An increment of PM_2.5_ levels increases the risk of asthma exacerbation resulting in emergency department visits, hospitalizations, and admissions to intensive care units, especially in a sensitive population such as small children [13,14,15,16]. The economic burden of uncontrolled asthma, including direct medical costs (hospitalization, emergency room visits, diagnosis test, and medications) and indirect medical costs (school absence and decreased productivity at work or school), are other aspects to consider, especially in low-income countries [17,18,19].

Thailand is a developing country in which WHO has estimated that the cost of 50 000 air pollution-related deaths in 2013 was about 60 billion US dollars [20]. Nowadays, the 24- hour average of PM_2.5_ levels of Thailand Ambient Air Quality Standards (NAAQSs) is 50 mcg/m^3^ for a healthy population, which is double the WHO recommendation (25 mcg/m^3^) [21]. The national ambient air quality standards (NAAQS) were first issued in Thailand in 1995, and the regulation was extended to PM_2.5_ in 2010. They were based on the law enforced by the Pollution Control Department (PCD) [22,23]. Although these have been issued for a decade, the problems have worsened, especially in growing major cities like Bangkok and Chiang Mai, where the drive for economic growth and environmental protections are in opposite directions. Therefore, we preferably look forward to having a successful Clean Air Act to lower the daily ambient air quality standard of PM_2.5_ concentrations from 50 µg/m^3^ to 25 µg/m^3^ (WHO guideline levels) to provide a better quality of life and protect from premature deaths in Thailand [24].

Like any other children worldwide, Thai children also deserve their rights, including being protected from any health issues caused by poor air quality. However, the national data about PM_2.5_ and health impacts are lacking. There were only a few studies among the Thai population regarding air pollution and asthma since we have limited resources for PM_2.5_ detection with only 123 stations for the whole country. Most of the stations are located in Bangkok Metropolitan Region and Chiang Mai province. Both cities have been recognized as the cities of the highest PM_2.5_ concentration in the country for decades.

To urge the Clean Air Act, it is essential to have an accurate measure of PM_2.5_ exposure and its association with the health problems in Thai children with asthma. Our study aimed to investigate the association between PM_2.5_ exposure and asthma exacerbation in children living in Bangkok Metropolitan and Chiang Mai region. Our study was conducted during the Covid-19 outbreak; and similar to many countries globally, Thailand implemented a lockdown policy starting from January 2021. The effect of the lockdowns will be taken into consideration.

## Methods

A pilot prospective cohort observational study was conducted from June 2020 to February 2021 in Bangkok, the vicinity, and Chiang Mai, Thailand. Seventy-two children from the Chest clinic and Allergy clinic at Ramathibodi Hospital, Mahidol University, Bangkok and from the Chest clinic at Nakornping Hospital, Chiang Mai; aged between 5 – 18 years who had been physician-diagnosed with asthma were included. In addition, residential address, demographic data; sex, age, height, weight, environmental factors, and asthma status since enrollment, was collected for each participant.

The Written Asthma Action Plan (WAAP) and a peak flow meter were given to all participants. There were routine follow-ups at the Chest or Allergy clinic every 1–3 months. The caregivers recorded the participant’s daily respiratory symptoms (cough and dyspnea), peak expiratory flow rate (PEFR) levels, asthma exacerbation (reliever use, school absences, ER visits, and hospitalizations) in the data record form within the WAAP; then reported to the researcher every month.

This study was approved by the Human Research Ethics Committee, Faculty of Medicine Ramathibodi Hospital, Mahidol University (COA. MURA2020/600), and had written informed consent from the legal guardians of all participants.

*Definition of well controlled asthma, severe asthma and asthma exacerbation are as of the followings* [25]; **Well controlled asthma** is defined as when the patient has no daytime asthma symptoms more than twice per week, has no night waking due to asthma, has no limitation of daily life activity and does not need any reliever more than twice per week. ***Severe asthma*** requires step 4–5 treatment (medium dose ICS-LABA or high dose ICS with a second controller) with poor symptoms control. **Asthma exacerbation** is the small airway obstruction that presents with cough, chest tightness or wheezing, or 20% declined PEFR which can be relieved by short acting beta-2 agonists.

### PEFR monitoring

Daily measurements of PEFR were measured with a Mini Wright peak flow meter™ (Clement Clark International Limited, London, UK) at the participant’s home twice a day (morning and evening). Both PEFR measurements were done before any medication was taken. Each test consisted of 3 maneuvers, and participants were instructed to record the largest PEFR readings in the WAAP.

### Assessment of daily ambient PM_2.5_ exposure level

Geocoding of the nearby location of participants’ residences were manually performed on google map by one researcher (KC). Their geographic coordinates in latitude and longitude pair were then obtained for point mapping and interpolating daily PM_2.5_ exposure in ArcGIS® Pro (Environment System Research Institute Inc., Redlands, CA, USA). The average daily ambient PM_2.5_ concentration and location of air monitoring station were obtained from the website of the Pollution Control Department (*http://air4thai.pcd.go.th/webV2/*) and the Climate Change Data Center of Chiang Mai University (*https://www.cmuccdc.org/*). The PM_2.5_ level from both sources was measured by the identical method using the continuous automated air sampling monitory station (Beta Radiation Attenuation and Tapered Element Oscillating Microbalance; TEOM) located in the residential area. The daily ambient PM_2.5_ levels were then interpolated by inverse distance weighted (IDW) method in ArcGIS® Pro (Environment System Research Institute Inc., Redlands, CA, USA) to determine the individual exposure at the residence of each participant during the study period (June 2020 to February 2021).

Several time-series studies have found lagged effects of PM_2.5_ on asthma symptoms (Lag days). The delayed symptoms are because PM_2.5_ induces free radical producing, imbalanced intracellular calcium homeostasis which activates inflammation in the lung [26]. The results of Lag days differed from 2 to 7 days [26,27,28,29].

### Statistical analysis

Descriptive statistics were conducted for all variables. Chi-squared was used to assess differences in proportion by group (p-value < 0.05 indicates a statistically significant difference). The association between PM_2.5_ level and asthma exacerbation was analyzed using Pearson correlation and regression models. The IBM® Statistical Package for the Social Sciences (SPSS®) version 23 for Windows was used for data analysis. The level of significance was set as 5%.

## Results

We excluded two the 72 participants (one had received immunotherapy during the study period, and the other had lost follow-up), leaving 70 participants where 67.1% were boys. The average age was 9.7 years (minimum 5.3 years old and maximum 18 years old). Forty-one participants (58.6%) had well-controlled asthma. Severe asthma was found in more than half of the participants, as shown in ***Table 1***.

**Table 1:** Descriptive statistics for 70 asthmatic children.


VARIABLES		ALL PATIENTS (%)

**Age (years)**		9.7 (5.3, 18)

**Gender**	Male	47 (67.1)

	Female	23 (32.9)

**OPD**	Chest Clinic (Bangkok)	39 (55.7)

	Allergy Clinic (Bangkok)	18 (25.7)

	Chest Clinic (Chiang Mai)	13 (18.6)

**Comorbidity**	Allergic Rhinitis	38 (54.3)

	Atopic Dermatitis	2 (2.9)

**Level of asthma controlled**	Well Controlled	41 (58.6)

	Partly/Uncontrolled	29 (41.4)

**Severity of asthma**	Mild	16 (22.9)

	Moderate	16 (22.9)

	Severe	38 (54.3)

**Environmental factors**	Passive Smoking	14 (20)

	Pets	23 (32.9)

	Air Purifier	26 (37.1)


**Figure 1:**
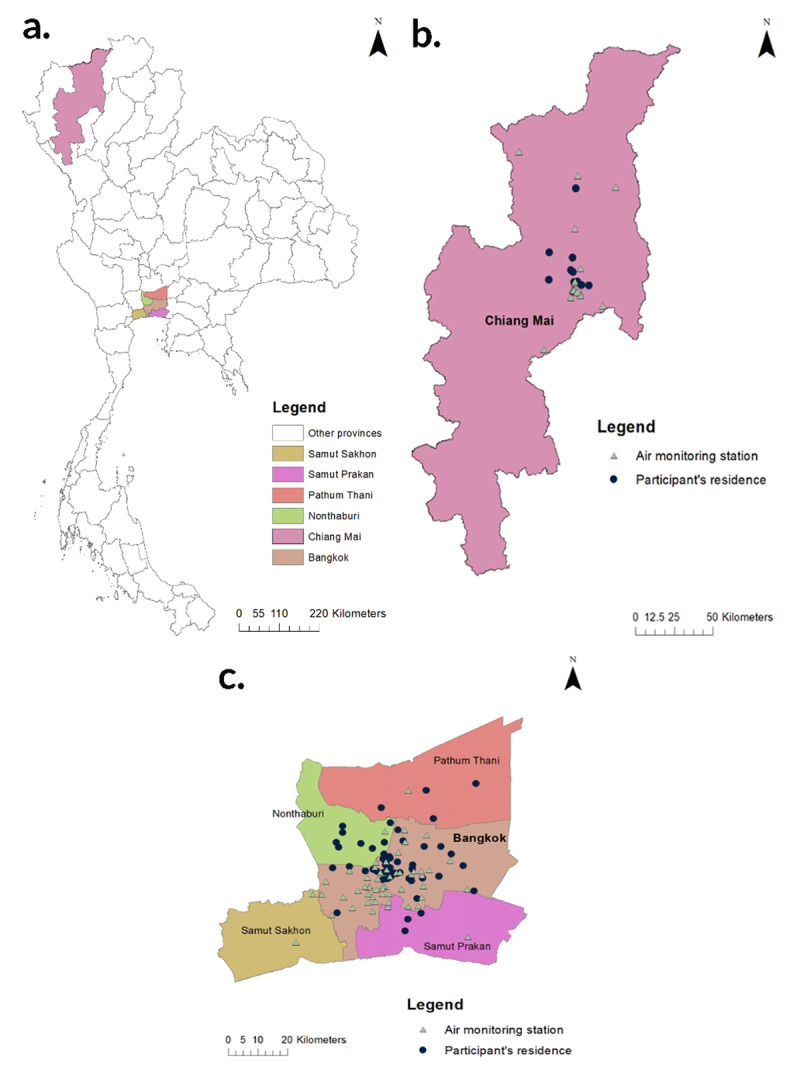
Study area in overview (A), the location of air monitoring station and residence of participants in each study site (Ramathibodi Hospital and Nakornping Hospital) are illustrated in B. and C. respectively.

**Figure 2:**
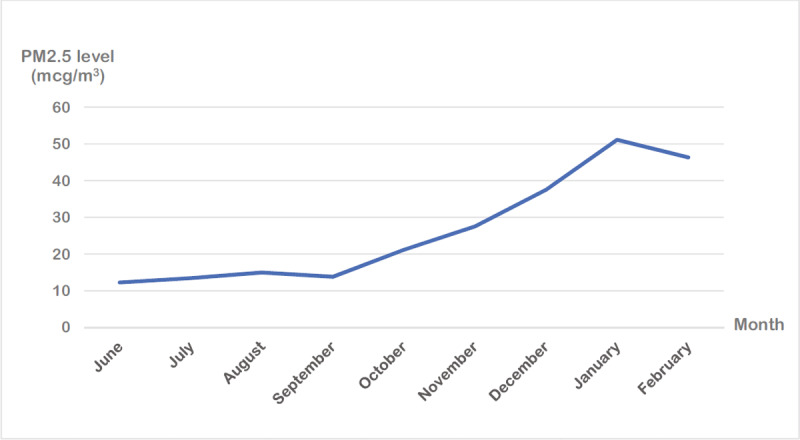
The average individual PM_2.5_ exposure.

The geographical distribution of participants’ residences and air monitoring stations is shown in ***Figure 1***. There were 96 air monitoring stations available to obtain the daily PM_2.5_ levels across Bangkok metropolitan Region (72 stations) and Chiang Mai province (24 stations) regarding participants residence during the study period. The results revealed that the average individual PM_2.5_ exposure at the residence in this study was elevated from October and reached the highest in January 2021, both in Bangkok and Chiang Mai (***Figure 2***).

**Figure 3:**
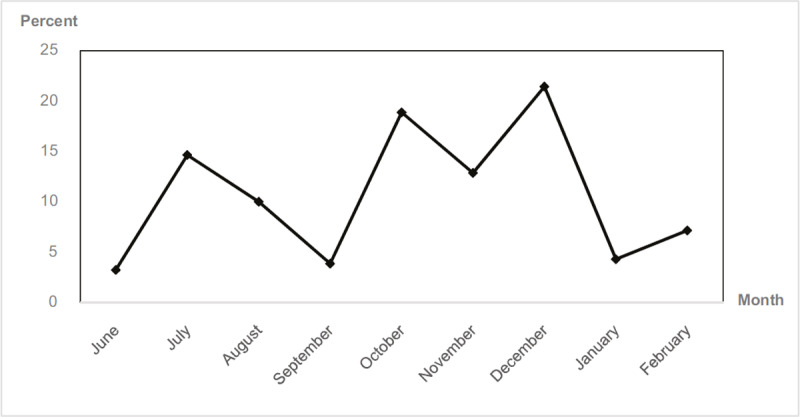
Incidence of asthma exacerbation among participants during the study period (June 2020 to February 2021).

The incidence of asthma exacerbation (dyspnea, reliever used, school absence, 20% declined of PEFR, ER visit, and hospitalization) is shown in ***Figure 3***. Thirty-two participants of 70 had exacerbated events during the study period (9 months), with the maximum in December (15 children had asthma exacerbation). The asthma status before enrollment was a significant risk factor of asthma exacerbation (uncontrolled group Vs. controlled croup = 89.7% Vs. 14.6%, p-value < 0.01) (***Table 2***). Age was associated with asthma exacerbation (hazard ratio = 0.79, 95% CI 0.68–0.93). But PM_2.5_ concentration, gender, and air purifier use had no statistically significant association with asthma exacerbation (***Table 3*** Hazard ratios for asthma exacerbation).

**Table 2:** Factors affecting asthma exacerbation.


FACTORS		EXACERBATION N = 32 (%)	NO EXACERBATION N = 38 (%)	P-VALUE

Asthma status before enrollment	Control (n = 41)	6 (14.6)	35 (85.4)	<0.001

	Uncontrol (n = 29)	26 (89.7)	3 (10.3)	

Severity of asthma	Mild (n = 16)	6 (37.5)	10 (62.5)	0.69

	Moderate (n = 16)	7 (43.8)	9 (56.2)	

	Severe (n = 38)	19 (50)	19 (50)	

Air purifier used		12 (46.1)	14 (53.9)	0.95

Passive smoking		7 (50)	7 (50)	0.72

Pet		10 (43.3)	13 (56.6)	0.79


**Table 3:** Hazard ratios for asthma exacerbation.


FACTORS	HAZARD RATIO	95% CI	P-VALUE

PM_2.5_ level	0.99	0.95, 1.03	0.8

Age	0.79	0.68, 0.93	0.004

Female	1.45	0.7, 2.96	0.3

Air purifier used	0.88	0.42, 1.85	0.75


For the acceptable PM_2.5_ levels, the cut-off point of 12 mcg/m^3^ (US criteria) has higher sensitivity to detect asthma exacerbation compared to the Thai criteria (37 mcg/m^3^) (sensitivity 98.2% vs 32.1%).

**Figure 4:**
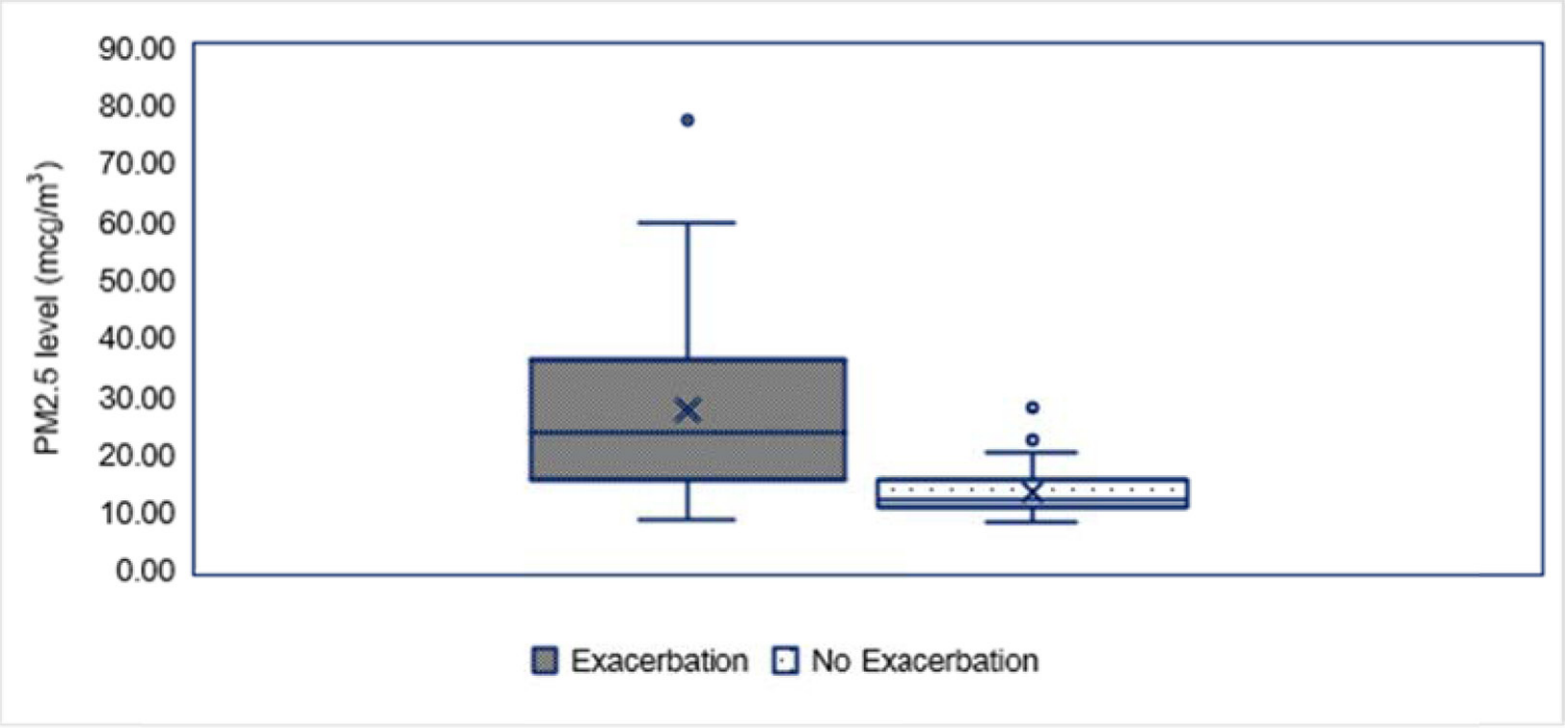
PM_2.5_ concentration and Lag Day 3 between exacerbation group and non-exacerbation group.

**Figure 5:**
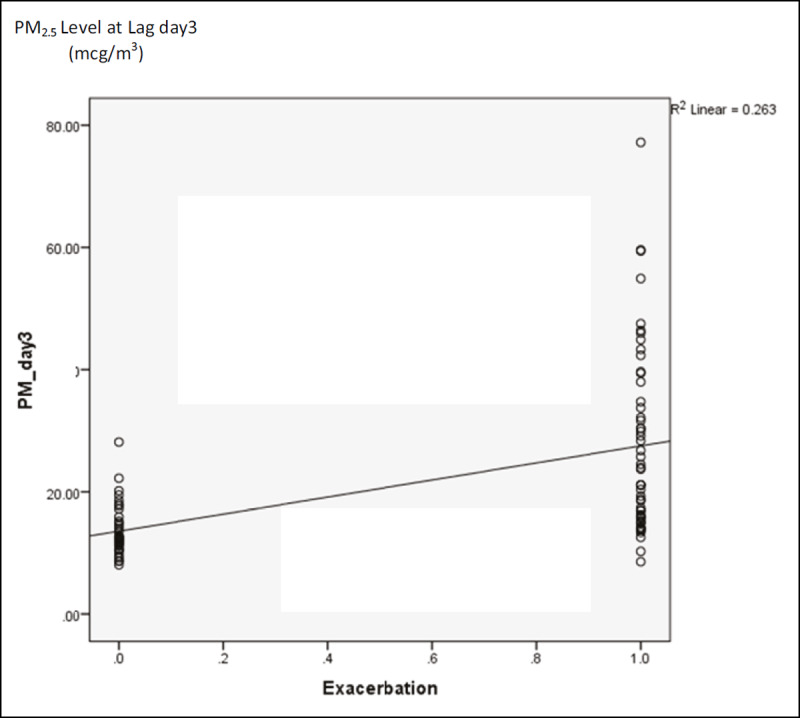
The association of PM_2.5_ increment and asthma exacerbation.

The PM_2.5_ level at 3 days before asthma exacerbation (lag day 3) was correlated with acute asthmatic attack (r = 0.62, p-value = < 0.01). Mean PM_2.5_ level exposure at lag day 3 in exacerbation and non-exacerbation group were 27.5 vs 13.6 mcg/m^3^ (p-value < 0.01) (***Figure 4***). Every 10 mcg/m^3^ of increment of PM_2.5_, there was a 0.2 event increase in asthmatic exacerbation (***Figure 5***).

Thirty-six patients completed the daily peak flow meter record; 16 of them had exacerbation (PEFR is less than 80% of baseline). Only one of these required hospitalization. All of them used reliever medication as directed in an asthma action plan.

## Discussion

Nowadays, we have many methods for estimation of PM_2.5_ levels, such as spatial interpolation methods, remote sensing techniques, air quality model methods, and machine learning methods [30]. This study calculated PM_2.5_ exposure levels from the nearest stations around the residential area and IDW interpolation for accuracy estimation. Spatial interpolation based on the IDW method of geographic information system (GIS) uses known sample data for calculated unknown data [31]. A previous study showed that estimating PM_2.5_ levels’ spatial distribution is best achieved using IDW interpolation [30]. For this reason, developing countries that have fewer PM_2.5_ concentration detectors can use this method for estimated PM_2.5_ levels exposure.

Thirty-two children had at least one asthma exacerbation (within 9 months) during the study. Our included had children with severe asthma up to 54.3%, higher than the normal asthma population [32] because Ramathibodi Hospital is a tertiary referral center.

Our study found an association between age and asthma exacerbation (hazard ratios = 0.79, 95% CI 0.68–0.93). Silverman RA et al conducted a study in New York City that revealed that age significantly affects hospitalization and ICU admission among asthmatic patients [16]. Compared to adults, children inhale a higher ratio of air mass per body weight. Therefore they are vulnerable to higher exposure to PM_2.5_ [19]. Also, asthma status prior to enrollment had a statistically significant effect on asthma exacerbation. Patients with uncontrolled asthma are more sensitive to air pollution and more difficult to treat.

Interestingly, our study revealed that the PM_2.5_ level at 3 days before the asthma symptoms occurred (lag day 3) was correlated with an acute asthmatic attack (r = 0.62, p < 0.01), while the PM_2.5_ concentration on the day of exacerbation was not statistically significant. Furthermore, the average daily PM_2.5_ level exposure at lag day 3 was significantly higher in the group of children with asthma exacerbation than those without any exacerbation. These lag effects of PM_2.5_ were previously demonstrated, and explained with regard to the process of inflammation and immune response [26,27,28,29,33]. Therefore, the hazard of PM_2.5_ in the sensitive patients could last for at least 3 days before an acute exacerbation occurs. The caregivers of children with asthma should be aware that personal protection, outdoor activities avoidance and monitoring of asthma symptoms and/or PEFR are recommended during the high season of PM_2.5_.

During the cold months (December to February), according to the database of the Pollution Control Department, Thailand [21], the monthly average PM_2.5_ concentration exceeded the Thai standard concentrations for sensitive patients as of 37 µg/m^3^. A previous study in Bangkok Metropolitan Region has shown that PM_2.5_ levels in the cold season were significantly higher [22]. During winter, the ridge from the high-pressure system along with the Northeast monsoon from China covered the Northern and the central region of Thailand. As a result, the cold and dry air containing air pollutants becomes stagnant and induces a radiative inversion. Without rain, the pollutants remain suspended in the air for a longer period [34,35].

Bangkok is in central Thailand. It is a crowded metropolitan area with high-rise buildings, industries, and complicated transportation, whereas Chiang Mai is in the Northern territories with mountains and agricultural fields. The sources of PM_2.5_ in the Bangkok Metropolitan area are usually traffic, industrial activities and open burning [34,36]. In contrast, in Chiang Mai, the primary sources are forest fires and biomass burning, such as crop field burning, especially sugarcane and rice. The burning season during the harvesting period runs from December to April [37].

The incidence of asthma exacerbation was increased in July and October to December 2020. In July 2020, children had high acute asthmatic attacks (14.6%), suspected to derive from a seasonal viral infection such as RSV, Rhino-enterovirus infection. However, as a low-income country, we had limitations in obtaining the viral study in all participants.

We discovered that the incidence of asthma exacerbation declined during the COVID-19 lockdown in Thailand, which occurred in January 2021. Previously, the concentrations of PM_2.5_ in Thailand reached a peak in December or January. The decline may derive from the disruptions of the children’s activities such as online learning, staying away from any respiratory viruses, and less exposure to outdoor air pollution. A study in 2020 concluded that the low traffic conditions during the lockdown resulted in improved air quality in Bangkok [38]. Recent studies, mostly from Asian countries such as India and China, revealed that the lockdowns have positively impacted air quality improvement [39]. The PM_2.5_ and PM_10_ concentrations were decreased globally during the lockdowns. The restrictions of transportation, travel, and social gatherings decreased fuel combustion, while the cessation of some industries led to the decline of air pollutants in the atmosphere.

Air pollution is responsible for at least 5 million premature deaths per year. The problem has increased rapidly; without intervention for these problems, the number of deaths due to ambient air pollution will double by 2050 [10]. In 2009 a study in the United States showed a decrease in PM_2.5_ exposure was associated with gains in life expectancy [40].

Air quality is one of the United Nations’ Sustainable Development Goals (SDGs) targets effectively from 2015 until 2030. Thailand’s NAAQS recommends that PM_2.5_ levels should not exceed 50 μg/m^3^ on a 24-hour basis. Unfortunately, the acceptable air quality is weaker than the WHO guideline levels and close to the unhealthy limit of 55 μg/m^3^ in the United States. The barriers to policy implementation include a lack of data from the health care sectors, intermittent public awareness regarding health impacts of air pollution, and uncontrolled human activities. As pediatricians, we have been trying to raise awareness of poor air quality affecting children, especially in vulnerable subjects such as asthmatic children. We hope that the results of this study provide essential data for improving the air quality standard in Thailand. Is it time to tighten our standard for our good health?

To reduce unacceptable PM_2.5_ levels, we should begin with more PM_2.5_ stations in all provinces. Recently, the number of pollution measurement stations has increased from 19 to 73 stations in Bangkok Metropolitan Region. The Clean Air Act policy has shown that the government and private sectors are concerned about air pollution. Concerning the significant sources of air pollution, the strict policies for the “Clean Air Act” should be implied in a combination of air quality regulation, energy and agricultural policies. In high population density, policies mimicking the lockdown such as “work from home” or “hybrid online/onsite learning” may be alternative ways to decrease traffic.

This study had a few limitations. Primarily, a small number of participants because of the COVID-19 pandemic. Secondly, using a written asthma action plan (WAAP) modified the ER visits and the rate of admission due to the appropriate use of the reliever and adjusted dosage of a controller as directed in the asthma action plan. And finally, the lack of indoor PM_2.5_ exposure data.

## Conclusion

When using a robust technique to estimate PM_2.5_ exposure in a residential area, the asthma exacerbations seem to increase in the high PM_2.5_ season. Children with asthma are sensitive to PM_2.5_ levels above 12 mcg/m^3^. Exposure to high PM_2.5_ levels can lead to asthma exacerbation within 3 days. Further study is needed for more recruitment to establish the national data.
